# A Multi-Component Model of Emotion Response Convergence: Implications for the Development of Psychopathology

**DOI:** 10.1177/17540739251335577

**Published:** 2025-08-06

**Authors:** Kalina J. Michalska, Dana E. Díaz

**Affiliations:** 8790 Department of Psychology, University of California Riverside, Riverside, California, USA

**Keywords:** internalizing symptoms, externalizing symptoms, emotional reactivity, emotion response convergence

## Abstract

A central tenet in emotion research is that emotional reactivity involves convergent changes across subjective, behavioral, autonomic, and more recently neural, response systems. This has led to a model where convergence across multiple response systems facilitates an individual's emotional responses to environmental stressors. However, empirical support for this model is mixed and limited work examines whether psychobiological convergence during emotional reactions unfolds differently among children at risk for psychopathology. In this paper, we review data examining whether atypical alignment between autonomic and subjective components of emotional reactivity is evident in youth with internalizing and externalizing problems. We contend that the direction and magnitude of psychobiological convergence may be meaningful for understanding individual differences in the development of psychopathology. We focus on autonomic and self-reported responses, commonly assessed in developmental work, and explore how their alignment may reflect meaningful variation in emotional experience. We propose that understanding the neural basis of this convergence could refine developmental models of emotion and inform early identification of risk. Finally, we outline methodological considerations for studying convergence across emotional systems in youth.

## Introduction

Most of us will readily recall a time when we felt afraid: during a particularly turbulent airplane trip or hearing a loud bang in the middle of the night as a child afraid of the dark. We may recall our heart pounding, our breathing quickening, and blurting out some form of anguished cry. Many models of emotion center around the presumed notion that emotions involve multiple coordinated components—autonomic, subjective, neurophysiologic, and behavioral (Damasio, 1998; Darwin, 1872; James, 1884, 1994; Lazarus, 1991; Levenson, 1994; Matsumoto & Ekman, 2009; Ortony et al., 1988; Sander et al., 2005). Yet, empirical support for strong *emotion response convergence* across these systems is mixed (Hollenstein & Lanteigne, 2014; Mauss et al., 2005; Quas et al., 2000; Quigley & Barrett, 2014), and few studies examine how emotion response convergence develops or varies in children at risk for psychopathology. Here, we focus on autonomic reactivity and subjective self-report, arguing that the direction and degree of their alignment may offer insight into individual differences in children's emotional functioning. We also propose that understanding the neural basis of autonomic responses and subjective reports can refine models linking emotion response convergence to developmental psychopathology, such as internalizing and externalizing symptoms. Given that (a) autonomic and subjective responses are the response modalities most often assessed in developmental studies, and (b) clinical evaluations often rely on self-reported autonomic states, we concentrate on these two measures. Finally, we discuss methodological considerations for interpreting response convergence across emotional systems in development.

## Emotion Response Convergence

The notion of emotion response convergence (sometimes phrased as emotion response coherence, emotional convergence, or synchronization), or the alignment of task-related changes in autonomic, behavioral, and self-reported subjective indices of emotional reactivity, features prominently in early theories of emotion (Cannon, 1927; Darwin, 1872; Frijda et al., 1989; James, 1884, 1994), as well as more contemporary models (Damasio, 1998; Evers et al., 2014; Matsumoto & Ekman, 2009; Sander et al., 2005; Scherer, 2009). For instance, a typical fear response like those chronicled above is thought to be a coordinated combination of elevated autonomic arousal, fearful facial expression, threat appraisal, and the desire to flee the threatening situation. In these theories, fear and other emotions like anger or disgust are conceptualized as a surge in the emotion system's subcomponents that motivate humans to respond adaptively to their current circumstances (Hollenstein & Lanteigne, 2014). One prominent emotion theory, the Component Process Model (Scherer, 1984, 2009), proposes that the cognitive evaluation of an event initiates the changes in autonomic and somatic systems and thus drives the emergence of convergent response patterning among all the emotion components (see also Dan-Glauser & Scherer, 2008).

## Variability in Convergence Across Emotional Systems

Empirically, there is significant variability in the alignment between emotional response systems (Lougheed et al., 2021; Michalska et al., 2013, 2022), with many studies observing weak or no convergence across indicators (e.g., De Los Reyes et al., 2012; Kreibig, 2010; Mauss et al., 2005, 2009; Siegel et al., 2018). Some approaches consider convergence as only loosely coupled (Lang, 1988) or limited to intense emotions (Brown et al., 2020; Russell, 2003; Scherer, 1984). Other scholars argue that divergence may be the norm rather than the exception due to emotion regulatory mechanisms that disrupt emotional processes (Butler et al., 2014; Dan-Glauser & Gross, 2013; Lanteigne et al., 2014). Despite these caveats, we contend that even though assessing emotion response convergence is inherently a complex and noisy science, it can nonetheless yield valuable insights into child and adolescent mental health. We review the literature showing that emotion response convergence across autonomic and self-reported responses may differ between youth with and without internalizing and externalizing symptoms. In sections to follow, we (1) outline key components of the autonomic nervous system (ANS) and central nervous system (CNS) involved in controlling autonomic responses and subjective self-reported emotions, (2) examine emotion response convergence across ANS activity measures, (3) consider how impaired ANS coordination during development confers vulnerability to broadband psychopathology categories (internalizing and externalizing disorders), and (4) assess emotion response convergence across autonomic and subjective self-reported emotions to identify alterations linked to the development of psychopathology. We describe patterns of internalizing and externalizing symptom expression, including hyper-awareness (heightened self-report accompanying typical levels of autonomic arousal); hypo-awareness (blunted self-report accompanying typical levels of autonomic arousal); and hypo-arousal (lower autonomic arousal accompanying typical levels of self-report). We believe such an integrative approach may enhance our understanding of developmental risk for psychopathology and guide treatment targets, such as skills to enhance emotional clarity, awareness of arousal-related autonomic cues, and novel real-time feedback approaches.

## Autonomic Measures of Emotional Responses and Their Neural Substrates

**Figure 1.**
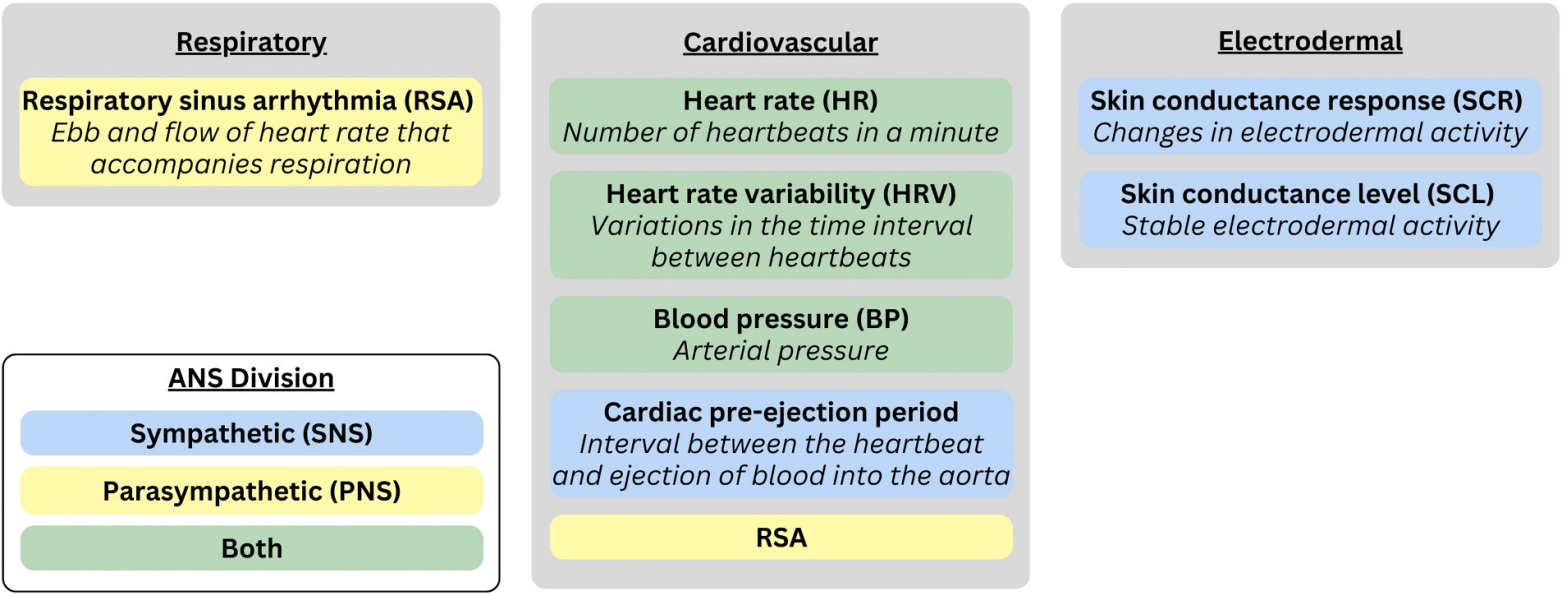
Commonly assessed autonomic nervous system (ANS) measures.

The ANS plays a vital role in influencing individual differences in the intensity and duration of emotional experiences (Levenson, 2003, 2014; Michalska & Davis, 2019; Öhman & Wiens, 2003). It consists of two coordinated branches that jointly support adaptive functioning: the sympathetic nervous system (SNS), which mobilizes the body in response to threats or challenges (Seery, 2011), and the parasympathetic nervous system (PNS), which promotes rest, social engagement and anticipatory regulation in safe contexts (Kemeny, 2003; Porges, 2009; Schulkin & Sterling, 2019). Importantly, the ANS is not a unidimensional system in which the activity of the SNS and PNS are universally reciprocal. Rather, depending on individual differences and contextual factors, the two branches can shape autonomic responses via multiple forms of coordinated and uncoordinated activity (Berntson et al., 1991; Gatzke-Kopp et al., 2020; Kreibig, 2010). In the following sections, we characterize multiple metrics of ANS functioning and provide evidence that each ANS branch can be activated independently, meaning researchers cannot necessarily infer SNS tone from PNS tone and vice versa (Berntson & Cacioppo, 2007). Thus, autonomic assessments that incorporate both SNS and PNS metrics provide a more comprehensive characterization of ANS functioning and further our understanding of developmental psychopathology (see Figure 1).

Frequently assessed indices of ANS activity in emotion research are obtained from cardiovascular, respiratory, and electrodermal (i.e., sweat gland) responses. Cardiovascular measures are quantified via heart rate and heart rate variability, respiratory sinus arrhythmia (RSA), cardiac pre-ejection period (PEP), and blood pressure (Mauss & Robinson, 2009). Electrodermal responses are quantified via skin conductance level (SCL) or momentary skin conductance responses (SCRs; Michalska et al., 2019). Each ANS measure differs concerning whether it primarily reflects SNS activity, PNS activity, or both. For instance, SCR and cardiac PEP predominantly reflect SNS activity; heart rate variabilityand RSA have been mainly associated with PNS activity (Larsen et al., 2008; Mauss & Robinson, 2009); and heart rate and blood pressure reflect a combination of SNS and PNS activity. To constrain the scope of our review, we focus on autonomic indicators tapping moment-to-moment arousal (e.g., heart rate, heart rate variability, RSA, and SCR), rather than tonic measures (e.g., SCL, baseline RSA), which provide a relatively non-specific measure of arousal across a longer window of time.

*Heart Rate, Heart Rate Variability, and Respiratory Sinus Arrhythmia.* The heart is regulated by both SNS and PNS branches of the ANS. The SNS predominantly influences stroke volume and contractile force (ionotropic), whereas the PNS modulates heart rate (chronotropic). Increases in heart rate may result from heightened SNS activity, reduced PNS activity, or both. Heart rate variability—the variation in time between two consecutive heart beats—serves as a noninvasive marker of ANS function (Critchley et al., 2003). RSA, a specific measure of heart rate variability within the respiratory frequency range, reflects rhythmic changes in heart rate tied to breathing (Beauchaine, 2001, 2015). Thought to index vagal activity (Porges et al., 1994), and therefore the PNS (but see Grossman & Taylor, 2007; Ritz, 2009, for caveats), RSA emerges from the interaction of cardiovascular and respiratory systems. During stress or challenge, PNS influence over the heart decreases, releasing a physiological “brake” to enable adaptive responses (Porges, 1995, 2007). In low-risk individuals, greater RSA reactivity is linked to positive affect and better emotion regulation (e.g., Blandon et al., 2008; Calkins & Keane, 2004; Davis et al., 2016). However, while moderate RSA withdrawal (i.e., decreases in RSA from baseline) can be adaptive, excessive withdrawal is associated with emotion dysregulation and psychopathology in both children and adults (Beauchaine, 2015; Beauchaine et al., 2019).

*Central nervous system control of cardiovascular function*. Accumulating research indicates that a network of CNS regions regulates cardiovascular activity, such as heart rate variability and RSA, during emotional experience (Mulcahy et al., 2019; Thayer et al., 2012; Thayer & Lane, 2000, 2009). Central to this system is the vagus nerve, which primarily transmits afferent signals (∼80%) from the heart and other organs to the nucleus tractus solitarius in the brainstem (Kalia & Mesulam, 1980; Porges, 2007, 2009). The nucleus tractus solitarius relays these signals to the reticular formation and, via the parabrachial nucleus (PB) and locus coeruleus (LC), to the forebrain. Importantly for emotional responding, the PB/LC complex projects widely, including to the hypothalamus and several thalamic regions that control the insular cortex, orbitofrontal cortex, and ventromedial prefrontal cortex (PFC) (Grady et al., 2020)—regions involved in inhibiting threat responses and retrieving safety memories (Glenn et al., 2020; Michalska et al., 2019). It also connects directly to the basolateral and central nuclei (CeA) of the amygdala (Gu et al., 2020; Kravets et al., 2015), key structures in emotional learning (Davis & Whalen, 2001) and adaptive emotional responding (Fox et al., 2015). These pathways enable the brain to monitor the body's internal state and adjust organ function accordingly.

Sensory signals from the body and viscera enter the nucleus tractus solitarius and are integrated with top-down inputs from other CNS regions, allowing cortical regions to modulate brainstem cardiorespiratory functions. A regulatory network involving the prefrontal, cingulate, and insular cortices activates the CeA, which in turn inhibits vagal motor neurons via the nucleus tractus solitarius (Beauchaine, 2015). This pathway translates PFC activity into RSA (Thayer et al., 2012). The PFC is key to executive functions, including emotion regulation (Etkin et al., 2015; Fuster, 2015; Kolb & Whishaw, 2009; Ochsner & Gross, 2007; Wager et al., 2008). PFC dysfunction, common in developmental psychopathology (White et al., 2017), is often linked to low resting RSA and exaggerated RSA reactivity, both markers of poor executive function (Graziano & Derefinko, 2013). Thayer's *Neurovisceral Integration Theory* (Thayer et al., 2009, 2012) suggests RSA reflects PFC function and thus indirectly indexes central emotion regulation mechanisms, supported by inhibitory medial PFC-to-PNS pathways and RSA-PFC correlations in functional magnetic resonance imaging (fMRI) studies (Beauchaine, 2015; Thayer et al., 2012).

*Skin Conductance Response.* Whereas heart rate, heart rate variability, and RSA are common in the stress and emotion regulation literature, SCR is widely used in studies of threat and fear learning. Skin conductance, or electrodermal activity, which reflects changes in eccrine sweat gland (located on the palms of the hands and soles of the feet) activity that alters the skin's electrical conductivity, is a direct index of SNS activation driven by cholinergic neurotransmission (Boucsein, 2012; Shields et al., 1987). In child research on emotion response convergence, SCR is more commonly assessed than SCL, especially when examining links to psychopathology. The most common SCR metric is amplitude, peaking 4–6 s after stimulus onset, making it a relatively slow response compared to RSA. SCRs are triggered by stimuli that are novel, intense, or emotionally evocative (Boucsein, 2012) and are associated with behavioral inhibition and avoidant coping—both relevant to childhood anxiety (Degnan & Fox, 2007; Obradovic & Boyce, 2012). Skin conductance is also influenced by appraisals such as goal relevance (van Reekum et al., 2004), aligning with Scherer's Component Process Model (Scherer, 2009), and linking cognitive evaluations to autonomic reactivity.

## Neural Underpinnings of Autonomic Function

Neuroimaging techniques like fMRI have clarified how the CNS supports autonomic responses to emotional stimuli. Studies often use affective tasks, like fearful faces or threat cues, to assess changes in peripheral physiology and model brain-body dynamics (e.g., Eisenbarth et al., 2016; Knight et al., 2005; Michalska et al., 2019). While autonomic responses are typically linked to the brainstem, subcortical structures like the amygdala (Büchel et al., 1998; Phelps et al., 2001; Williams et al., 2001, 2005), and cortical areas like the anterior insula (Büchel et al., 1998), anterior cingulate cortex (ACC) (Critchley et al., 2003, 2011; Williams et al., 2005), and dorsolateral and ventromedial PFC (Abend et al., 2020; Büchel et al., 1998; Critchley et al., 2000; Critchley & Harrison, 2013; Fredrikson et al., 1998; Michalska et al., 2019; Mullins et al., 2024; Nagai et al., 2004; Phelps et al., 2001; Tranel & Damasio, 1994; Williams et al., 2001, 2005), also play key roles in autonomic regulation. The insula, for example, supports autonomic regulation (Diserens et al., 2006) and represents internal bodily states (Craig & Craig, 2009; Critchley et al., 2004), especially in responses to interoceptive threat signals, such as a racing heart. Alongside the ACC, it helps translate bodily signals into emotional experience (Craig, 2002, 2003; Damasio, 2019). The ACC, a crucial interface between the limbic system and PFC (Bush et al., 2000; Craig & Craig, 2009), contributes to somatic regulation and has been linked to cardiovascular arousal during effortful tasks (Critchley et al., 2000) and SCRs to in children and adults (Michalska et al., 2019; Milad et al., 2007). Evidence also suggests that the dorsal ACC supports subjective emotion (Bush et al., 2000), while rostral anterior and anterior midcingulate regions are involved in implicit emotion processing (Bush et al., 2000; Lane, 2000). Dysregulated cingulate activity has been implicated in internalizing problems (IPs) such as depression and anxiety (Davidson et al., 1999; Peyron et al., 2000). We contend that these neural-level insights offer valuable perspectives on emotion response convergence, particularly in development.

## Autonomic Response Convergence

Despite its complexity, measuring multiple ANS signals, like heart rate, RSA, and SCR, can reveal the autonomic foundations of emotional reactivity. Problematically, researchers often treat these signals as interchangeable and examine the SNS and PNS branches separately, assuming convergence between systems and inferring one branch's activity from the other (Stone et al., 2020). Of note, although some overlap exists (e.g., Wong et al., 2007; Ziegler et al., 2009), distinct neural pathways control different organs, enabling bidirectional central-autonomic communication (Eisenbarth et al., 2016). Additionally, differences in response latency, rise time, rate of change, and recovery across modalities (Cuve et al., 2023) further complicate interpretation. Few studies examine SNS-PNS coordination, even though such integration may better indicate mental health concerns, like internalizing and externalizing symptoms, than studying each system in isolation (e.g., Berntson et al., 2008; Rudd & Yates, 2018; Thayer et al., 2010). In the next section, we review extant empirical work indicating that measures of ANS activity do not always hang together and discuss how emotion response divergence may be meaningfully associated with psychopathology symptoms in childhood.

Emotion research often assumes that autonomic systems must always “hang together” and respond in unison—e.g., heart rate and SCR increasing together (Grandjean et al., 2008; Valenza et al., 2012). But is that always the case? Correlations across autonomic measures are typically modest (Mauss et al., 2005), likely because heart rate reflects both SNS and PNS activity, SCR is SNS-specific, and RSA reflects PNS input. Though historically seen as working in opposition, the SNS and PNS are anatomically independent, so the function of one cannot reliably predict the other (Gatzke-Kopp et al., 2020; Gatzke-Kopp & Ram, 2018; Stone et al., 2020).

Emergent research has begun examining how the SNS and PNS branches coordinate. Berntson and colleagues’ (1991) *Doctrine of Autonomic Space* outlines four patterns: reciprocal (inverse activity), coactive (both increase), coinhibitory (both decrease), and independent. Whereas lower level reflexes tend to be reciprocal, higher order cognitive processes can produce any of these coordination types (Berntson et al., 1991, 1994; Berntson & Cacioppo, 2007; Gatzke-Kopp et al., 2020).

Convergence between autonomic signals depends on whether measures are derived from the same organ (e.g., RSA and cardiac PEP) or different ones (e.g., RSA and SCR). Even within a single branch, outputs to different organs can be independent (Berntson et al., 1991; Berntson & Cacioppo, 2007; Kreibig, 2010; Norman et al., 2014). For instance, antagonists block increases in heart rate but not SCR during stress (Jacobs et al., 1994), and stress can activate some muscle sympathetic nerves while leaving others unchanged (Wallin & Fagius, 1988). Thus, sympathetic activity can be organ-specific (Berntson et al., 1998), and measures may not always align. These foundational studies remind us that because ANS signals reflect distinct physiological processes, a multi-system approach—assessing cardiovascular, respiratory, and electrodermal responses—provides a more complete picture of ANS functioning in emotion (Kreibig, 2010). Although eccrine glands and cardiac systems share some neural control when measures of both systems change from baseline (Critchley, 2005), evidence shows largely distinct neural patterns predict cardiac and SCR responses under stress (Eisenbarth et al., 2016), indicating their different CNS origins. To better characterize convergence between autonomic systems and between autonomic and subjective responses, researchers should, when feasible, measure multiple forms of autonomic activity. Moreover, because RSA and SCR are shaped by both central and peripheral systems (Critchley, 2002)—and these systems undergo developmental change (Dollar et al., 2020; Giedd et al., 1999)—studies of emotion response convergence must also consider neurodevelopment, a topic we turn to next.

## Developmental Patterns of Autonomic Response Convergence

Studying emotion response convergence in adults is already formidable challenge, but in developmental affective psychobiology, the complexity is exponentially greater: the ANS is developing, the CNS is developing (see Gogtay et al., 2004 Movies 1–4 for a visual depiction of CNS development progressing from brain stem and subcortical structures to posterior and then anterior cortical structures), and subjective feelings—conscious emotional states that a child identifies and labels—are developing. Further complicating matters, the interactions between physiology, brain function, and self-report are also in flux. Emotions are shaped by multiple, bidirectionally interacting levels of autonomic activity (Hastings et al., 2014), and these interconnections shift over time (Larsen et al., 2008; Miskovic & Schmidt, 2012). Unsurprisingly, most emotion research on the integration of ANS with subjective experience has focused on adults, with limited studies exploring concurrent autonomic and subjective responses in children (El-Sheikh et al., 2009; Fortunato et al., 2008). These studies often yield mixed results: some studies find convergence between autonomic and subjective responses (Buss et al., 2004, 2005; Calkins & Dedmon, 2000; Talge et al., 2008), while others report no associations or inverse associations (Casey, 1993; Forbes et al., 2006; Hessler & Katz, 2007; Hubbard et al., 2004; Quas et al., 2000; M. Smith et al., 2011). Most rely on cross-sectional data, despite the fact that peripheral, neural, and cognitive systems co-develop and influence one another over time. A key task for developmental researchers is to uncover how early changes in one system shape the development of others (Hastings et al., 2014)—and ultimately how these dynamics shape subjective emotional experience.

Taking on this daunting challenge, a handful of investigators have begun using dynamic analytic strategies to better capture autonomic coordination across development and to examine whether some measures show greater early-life stability than others (e.g., Gatzke-Kopp et al., 2020; Gatzke-Kopp & Ram, 2018; Rudd & Yates, 2018; 2020). Understanding patterns of stability and change across systems offers insights into how autonomic function shapes subjective experience and emotion response convergence. In a longitudinal study of children 5–8 years, Gatzke-Kopp and Ram (2018) assessed autonomic coordination during passive film viewing using SCR and cardiac PEP as sympathetic indicators (the latter not reviewed here), and RSA as a parasympathetic measure. RSA showed strong trait-level stability across three annual visits, while SCR was more variable and driven largely by differences in momentary responses to stimuli, suggesting it reflects situational engagement rather than trait characteristics. Both RSA and SCR also exhibited developmental change across visits, highlighting the influence of developmental maturation on cardiac and electrodermal physiology. RSA increased nonlinearly over time, consistent with prior findings in children aged 4.5–7 years (Marshall & Stevenson-Hinde, 1998) and from infancy to 4 years of age (Bar-Haim et al., 2000). In contrast, SCR declined linearly (Gatzke-Kopp & Ram, 2018), possibly due to decreases in an early component of SCR thought to reflect attentional processes (Gao et al., 2007), and increasing habituation (Gao et al., 2007; Prokasy & Ebel, 1967). Cardiac measures showed greater rank-order stability at age 5–8 years than previously observed in infants and were comparable to findings in preschool (Calkins & Keane, 2004) and older children (age 6–13 years; El-Sheikh, 2005, 2007), and other samples within this age range (Marshall & Stevenson-Hinde, 1998). The low stability in SCR suggests individual differences in the decline rate and provides an opportunity to explore sources of within-individual change. Nonlinear variation across time may reflect cumulative stress exposure (Gatzke-Kopp & Ram, 2018) consistent with diathesis-stress models of psychopathology (Fletcher et al., 2019), early caregiving environments (Raine et al., 2002), or broader adversity-related factors (Gatzke-Kopp, 2016; Hinnant et al., 2013; Johnson et al., 2017), potentially implicating cortisol's influence on sympathetic activity. To better assess emotion response convergence in children, future studies should examine longitudinal trajectories and links between subjective self-report and autonomic measures across varied psychosocial contexts, given the significant amount of unique variance accounted for by developmental changes. Incorporating ecological momentary assessments of daily stressors and life events may further clarify how subjective stress and autonomic reactivity interact across development.

Developmental research on multi-system autonomic coordination reveals that in early childhood, the SNS and PNS are primarily reciprocally coordinated or jointly co-inhibited (Alkon et al., 2003; Gatzke-Kopp & Ram, 2018; Salomon et al., 2000). The scarce developmental data indicate that cross-system convergence increases with age, reflected in more consistent reciprocal activation patterns (Alkon et al., 2003, 2011, 2014), though some studies find no change between kindergarten and second grade (Gatzke-Kopp & Ram, 2018). These findings imply that maturation brings more integrated autonomic responses, enabling more effective regulation of arousal. Supporting this, dynamic modeling in 6-year olds shows reciprocal sympathetic activation (as indexed by PEP attenuation and RSA withdrawal) during a brief laboratory challenge task (Rudd & Yates, 2020), with preliminary evidence that changes in PEP temporally precede subsequent changes in RSA, suggesting that parasympathetic activity follows sympathetic activity during the task. Given individual variability in coordination trajectories, such modeling approaches may offer more nuanced insights into the developmental dynamics of ANS coordination to inform child psychopathology symptoms. There is substantial heterogeneity in the type and timing of coordination across autonomic response systems, a point we return to in the following section.

Emotional context may additionally influence the degree of autonomic coordination. Reciprocal coordination tends to be stronger during approach-oriented emotions (e.g., happy, angry) and weaker during avoidance-related states (e.g., fear, sad), likely due to the higher arousal and energy demands of approach behaviors (Gatzke-Kopp & Ram, 2018). Although the SNS and PNS typically function antagonistically, they can become uncoupled or co-activate/co-inhibit depending on environmental demands, emotional states, or atypical stress responses (Zisner & Beauchaine, 2016). Such patterns are crucial for understanding how individual differences in psychophysiology contribute to psychopathology risk (Beauchaine, 2001; Thayer & Lane, 2000).

## Emotion Response Convergence Between Self-Report and Autonomic Arousal

Research on the neural underpinnings of subjective experiences has progressed significantly (Dehaene & Changeux, 2011). This work suggests that subjective conscious experience emerges from non-conscious processes that enable cortical regions to re-represent lower order information, facilitating conscious awareness of one's environment (LeDoux & Pine, 2016). Subjective feelings of fear or anger, for example, are not direct products of subcortical neural circuits involved in defensive responses but rather arise from higher order circuits responsible for conscious experience, including the lateral and medial PFC, and parietal cortex, which are also critical for cognitive processes such as attention and working memory (Miller & Cohen, 2001). Understanding subjective experience in terms of intensity allows for an evaluation of how autonomic signals coordinate with subjective reports, providing insight into the phenomenological experience of emerging emotions and potential developmental trajectories related to psychopathology. While we primarily focus on intensity (in that we evaluate the coordination of autonomic signals with subjective experience and apprize the relative magnitude of one level of analysis compared to the other), prior research suggests that valence may also moderate the autonomic-subjective arousal relationship (e.g., Kreibig, 2010; Larsen et al., 2008; Siegel et al., 2018).

Studying emotion response convergence across development necessitates an understanding of the development of subjective self-report, which is influenced by interoceptive awareness—the ability to perceive fluctuations in internal physiological states (Damasio & Dolan, 1999; Dunn et al., 2010; Pollatos et al., 2005; Terasawa et al., 2013). Although direct research on this topic remains limited, prior findings suggest that developmental changes influence how autonomic and subjective self-reports align. Infants react emotionally before they can articulate emotions, displaying SNS activation and avoidance behaviors (Hane et al., 2008; Tronick, 1989, though see LoBue & Adolph, 2019). As language development progresses, it enables more complex appraisals of emotional experiences, shaping both expression and experience of emotions. Language development thus scaffolds the expression of internal emotional states and is linked to emotional awareness (Riggs et al., 2006; Saarni, 1999). The linguistic labels assigned to internal emotional experiences define those experiences, which, in turn, shape appraisals of autonomic signals (Doyle et al., 2021; Widen, 2013).

Unlike autonomic assessments, subjective self-reports are susceptible to social desirability influences (see e.g., Robinson & Clore, 2002). However, these influences may be less pronounced in younger children, as they have had less exposure to social norms shaping overt behaviors (Gnepp & Hess, 1986; Underwood et al., 1992). Although subjective self-reports are shaped by social, motivational, and contextual factors (Nederhof, 1985), these factors likely intensify with age. For example, in a study examining empathic responses in 4- to 17-year olds using subjective (behavioral evaluations), autonomic (pupil dilation), and hemodynamic (fMRI) measures, female participants reported feeling significantly more upset with age when witnessing another person in pain, whereas male participants reported feeling less upset with age (Michalska et al., 2013). Of note, autonomic and neurophysiological responses did not exhibit corresponding sex-related differences, suggesting a divergence between subjective self-report, autonomic, and hemodynamic responses. This divergence likely reflects the growing influence of social expectations, gender norms, and social learning on subjective self-report with age, whereas autonomic and neurophysiological systems remain relatively uninfluenced. In the absence of longitudinal data, however, this suggestion remains speculative. Examining emotion response convergence across multiple contexts while accounting for cultural and social factors will help refine models of how emotion response convergence develops in children. To move research in this area forward, studies should investigate cross-time emotion response convergence using multiple emotion elicitors, capturing subjective self-report and moment-to-moment autonomic assessments across key developmental periods. Characterizing the alignment between subjective self-report and autonomic profiles throughout development will provide insight into whether variations in convergence predict psychopathology risk. Advances in dynamic systems modeling provide new opportunities to inform such models by examining developmental and individual differences in the moment-to-moment manifestation of emotion response convergence (Rudd & Yates, 2020).

To disentangle whether emotion response divergence is a cause or a byproduct of psychopathology symptoms, researchers might examine whether autonomic coordination and emotion response convergence during early development (e.g., during the transition from kindergarten to preschool, a sensitive period marked by high PFC plasticity supporting autonomic regulation) are prospectively associated with changes in children's internalizing and externalizing symptom trajectories across childhood and adolescence. Emerging work on between-child differences in approach- and avoidance-specific autonomic coordination (i.e., SCR and RSA; Gatzke-Kopp & Ram, 2018) highlights the need for further longitudinal investigations of such epoch-to-epoch emotion dynamics across different timescales. Given the nonlinear nature of development, leveraging analytical strategies such as nonlinear growth modeling (e.g., generalized additive mixed models, latent curve models, or latent change score models) will enhance our ability to assess emotion response convergence across multiple developmental periods and at multiple time scales.

## Potential Moderators of Emotion Response Convergence

Situational factors may influence the extent to which children exhibit emotion response convergence. For instance, the valence of the experienced emotion and the discrete emotion itself may shape the degree of autonomic and subjective alignment between children. Convergence may be more pronounced for negative emotions, as distress can elicit stronger co-regulation processes (Feldman, 2012). Similarly, the specific emotion experienced (e.g., fear vs. anger) may differentially affect emotion response convergence, given that some emotions elicit more affiliative responses than others. While it is beyond the scope of our paper to consider experience at the level of discrete emotion, we refer readers to relevant work (e.g., Kragel & LaBar, 2013; Satpute et al., 2016; Siegel et al., 2018) to inspire future research on this dimension, such as whether some discrete emotions exhibit greater convergence than others, and whether this changes developmentally or as a function of psychopathology symptoms. Additionally, the social and situational context in which emotions are experienced, such as the presence of a supportive caregiver or a conflictual interaction, may further moderate convergence processes.

Beyond these situational influences, trait-level individual differences may also moderate emotion response convergence. For example, infant attachment relationships, such as attachment avoidance and attachment anxiety, play a role in shaping emotion regulation tendencies (Borelli et al., 2017; 2018). Attachment researchers posit that avoidantly attached infants, who have learned to expect rejection from caregivers, may suppress aversive arousal to minimize further rejection (Borelli et al., 2017; 2018; Cassidy, 1994). Empirical evidence supports this possibility, showing that children with avoidant attachment exhibit high autonomic arousal (i.e., neuroendocrine reactivity) but low self-reported distress in response to a laboratory stressor compared to secure children (Borelli et al., 2014). Conversely, anxiously attached infants, who experience inconsistent caregiving, may hyperactivate negative emotions to maintain caregiver responsiveness (Borelli et al., 2017, 2018; Cassidy and Berlin, 1994). Future research would benefit from longitudinal data collection using moment-to-moment autonomic assessments across infancy through adolescence to better differentiate predictors from correlates or consequences of emotion response convergence and divergence.

## Emotion Response Convergence and Psychopathology

Research on emotions and psychopathology indicates that emotion response convergence may be compromised when youth present with psychopathology symptoms, including both IPs and externalizing problems (EPs) (Hastings et al., 2009; Mullin & Hinshaw, 2007; Michalska et al., 2022). IPs are associated with greater anxiety, worry, sadness, and heightened neural reactivity in anticipation of ambiguous or uncertain threat (Michalska et al., 2023), whereas EPs are associated with greater experience and expression of anger but reduced fear and weaker cardiovascular reactivity (Beauchaine, 2001; Beauchaine & Thayer, 2015; Hastings et al., 2007, 2009; Raine et al., 1995). These patterns may reflect emotion dysregulation contributing to psychopathology, with disruptions in adaptive affective responses leading to maladaptive behavioral reactions.

**Figure 2.**
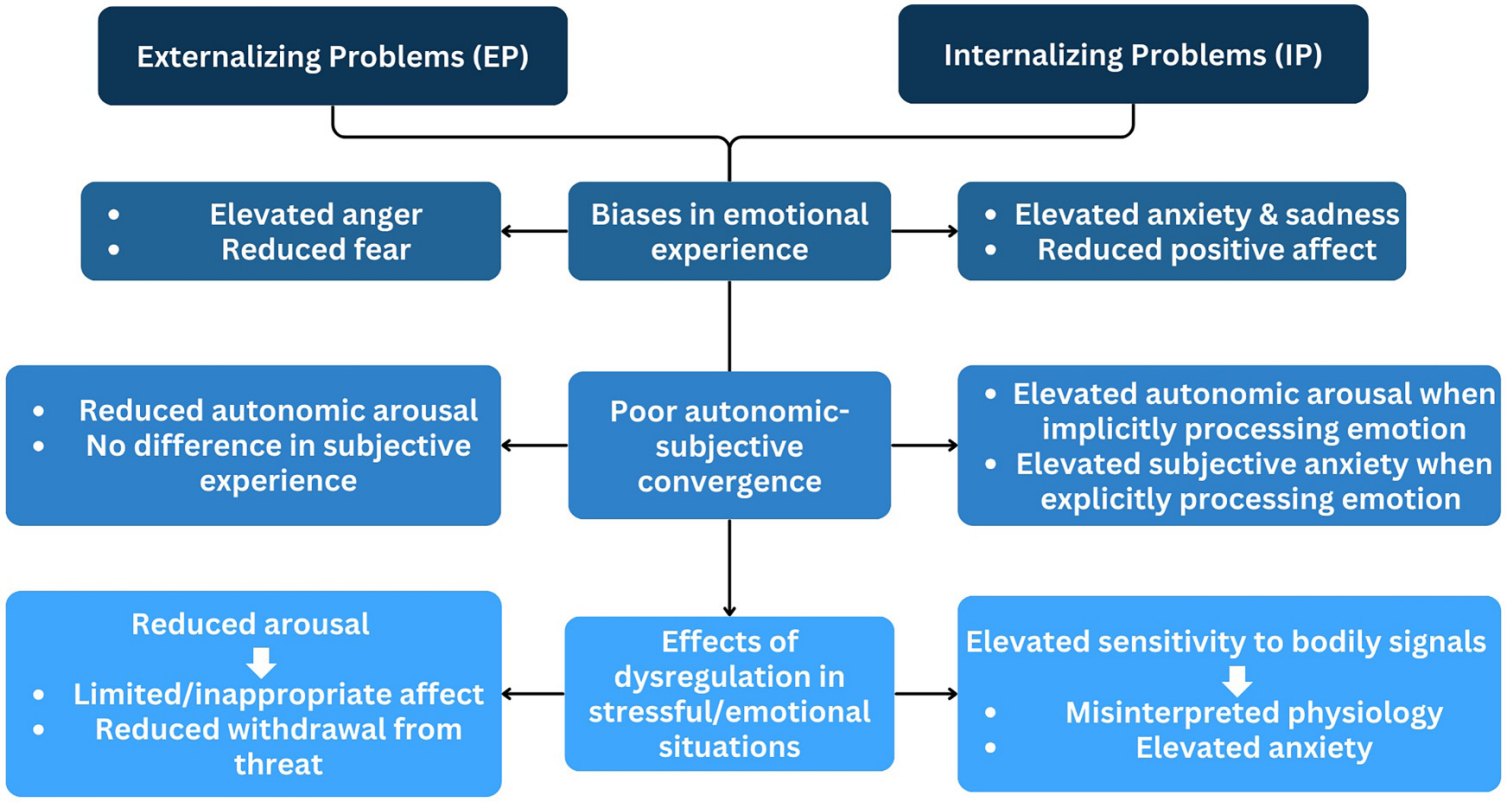
Theoretical model of emotion response convergence across internalizing and externalizing disorders.

In this section, we review data from our work and others examining whether atypical emotion response convergence between autonomic and subjective responses is evident in youth with IP and EP and highlight studies that incorporate neural data. Although few studies have directly investigated links between convergent responding and youth psychopathology (Lanteigne et al., 2014; Marsh et al., 2008), existing findings suggest that emotion response convergence holds promise for advancing our understanding of children's emotional development. We also argue for the inclusion of functional neuroimaging to help disentangle the contributions of various systems to emotion response convergence (see Figure 2).

### Internalizing Problems

IPs often present as sadness, anxiety, and worry—emotions partly rooted in subcortical brain regions that mature early in life. Individual differences in anxiety are linked to functional variation in the extended amygdala (Davis, 1992; Shackman & Fox, 2016), the septo-hippocampal system, and the ventromedial, ventrolateral, and dorsolateral PFC (Michalska et al., 2023). Anxiety is especially well-suited for delineating associations between emotion response convergence and psychopathology. Thinking back to the child who hears a loud noise in the dark and feels afraid in anticipation of something dangerous emerging, we might intuit a particularly tight coupling between their subjective anticipatory fear and their physiological responses like a racing heart and rapid breathing. Below we review the empirical evidence on emotion response convergence in anxiety, noting that most studies assess it indirectly, and few examine autonomic, subjective/perceived physiological, and neural responses simultaneously in children.

## Emotion Response Convergence in Anxiety

Evidence from studies comparing individuals with low- and high-trait anxiety confirms that those high in trait anxiety report greater anxiety in fear-inducing situations than those low in trait anxiety (Shechner et al., 2014). Of note, anxious individuals do not always exhibit heightened autonomic arousal (see Patterson & Ritts, 1997 for a meta-analysis). In one study, Mauss et al. (2004) observed that while anxious adults *reported* elevated autonomic activation (as measured by heart rate, SCR, blushing, and respiration), their *actual* autonomic responses during an anxiety induction did not differ from those of low-anxious individuals—suggesting hyper-awareness. That is, anxious individuals’ subjective emotional reports diverged from their autonomic responses, a pattern observed in other studies with anxious adults as well (Borkovec et al., 1974; Britton et al., 2013; Edelmann & Baker, 2002). We have recently extended these findings to children aged 8–12 (Michalska et al., 2022), examining the relationship between autonomic arousal and self-reported negative emotions in predicting children's IP. Results parallel some aspects of the adult findings and differ in others. Specifically, high-anxious children in our study exhibited high autonomic (SCR) and high subjective self-reported negative emotions in response to fear-inducing vignettes. That is, greater anxiety was observed only in children who displayed both elevated SCR and reported experiencing strong negative emotions (i.e., fear). Those with similarly high SCR but average or lower levels of self-reported negative emotions did not rate themselves as anxious. These findings align with cognitive models of anxiety, which emphasize threat evaluation biases, heightened self-focused attention, and atypical appraisal of social situations (Leary & Kowalski, 1995; Michalska et al., 2022; Mogg & Bradley, 2018). In particular, negatively biased appraisal (i.e., ascribing fear, sadness) of autonomic activation might play a key role within the cognitive processes implicated in anxiety. Our pattern also suggests increased psychobiological convergence among high-anxious children during middle childhood, reflecting alignment between subjective experience and physiological arousal (Michalska et al., 2022). While this differs from the above adult findings (Mauss et al., 2004) that emphasize a decoupling of emotional response systems in anxiety (i.e., heightened self-reported emotion accompanying typical or average levels of autonomic arousal), our results echo adult studies showing heightened convergence in specific contexts, such as phobic fear (e.g., Schaefer et al., 2014). Thus, rather than contradicting adult literature, these findings highlight that emotion response convergence may be context-specific, varying by type of anxiety, emotional stimulus, or developmental stage. Convergence might be more likely to emerge in response to discrete, fear-evoking stimuli than generalized anxiety cues—a possibility that aligns with distinctions drawn in adult research (e.g., Lang et al., 1998). We posit that anxious children and adults may be hyper-aware or overly attuned to their autonomic responding in stressful situations, potentially leading to cognitive distortions regarding what their autonomic states signify (Mauss et al., 2004; Michalska et al., 2022; Reed et al., 1990) and further amplifying anxiety symptoms (Paulus et al., 2019). Such hyper-awareness may indicate over-estimation of autonomic response by self-reported response (Michalska et al., 2022), which could become more pronounced with repeated instances of anxious experiences over time. Together these data suggest that different indices of emotional experience relate to one another in divergent ways in anxious *vs.* non-anxious children, with anxious children generally evidencing higher self-reported negative emotions (fear, sadness) relative to their autonomic responses. Overall, the findings underscore the need for a more nuanced view of emotion response convergence across developmental stages and anxiety subtypes.

One assumption underlying many psychological interventions, such as emotion-focused therapy (EFT), is that greater awareness of one's emotions ostensibly facilitates mental health (Smith et al., 2024). From this perspective, awareness occurs when one's subjective perceptions of one's emotional states align with what transpires in one's body. While this is sometimes beneficial, emerging data on emotion response convergence in IP suggests that overly high levels of awareness may, in some contexts, be detrimental to children's emotional functioning and potentially exacerbate anxiety (Michalska et al., 2022). Youth who are overly attuned to fluctuations in autonomic arousal may experience chronic discomfort, escalating and maintaining anxiety symptoms. They might turn to alcohol, other substances, or self-harm to alleviate negative somatic states (Borelli et al., 2018). Thus, understanding how implicit autonomic and explicit cognitive systems become less coordinated over time and potentially increase the risk for IP is of considerable importance.

Progress has stalled in our understanding of the role of emotion response convergence in childhood IP, as most studies rarely consider development. Autonomic systems underlying emotional arousal and downregulation (e.g., SNS, PNS) undergo developmental change, particularly during puberty (Gunnar et al., 2009). With age, children become more skilled at managing their emotions to achieve social goals, often masking undesirable emotional expressions (Rosenblum & Lewis, 2003) such as nervous laughter, sarcasm, and other forms of expression that are discrepant from underlying feelings (Flannery et al., 1993). How anxious children modulate their arousal and expressions can influence the frequency, intensity, and persistence of emotional responses normative for their age.

## Skin Conductance Response-Brain Convergence in Internalizing Problems

Neuroimaging findings refine our understanding of emotion response convergence in IP. For instance, the amygdala, a broad relevance detector, is reciprocally connected to various brain areas, including subcortical structures, allowing for affective influence on SNS and PNS regulation of cardiovascular activity, respiration, and electrodermal activity. While the amygdala plays a key role in autonomic learning aspects of emotion (Adolphs, 2013; Davis, 1992; Panksepp et al., 2011), evidence suggests that subjective emotional experience does not solely arise from this region (LeDoux & Pine, 2016; Winkielman et al., 2005). Patients with amygdala damage exhibit impairments in conditioned fear responses, fear-potentiated startle (FPS) responses, and arousal-enhanced perception and memory, but rarely show impairments in their subjective experience of emotion (Anderson & Phelps, 2002). This suggests that the amygdala's role in autonomic responses is relatively independent of mechanisms underlying subjective feelings.

Similarly relevant to IP, fMRI work using a developmentally appropriate threat conditioning paradigm reveals early-life social reticence temperament, characterized by shy, anxiously avoidant behavior, predicting a distinct pattern of hemodynamic-autonomic (as measured by SCR) convergence when recalling extinguished threat and safety cues (Michalska et al., 2019). Convergence was measured via amplitude modulation by trial-wise SCR, allowing examination of trial-by-trial associations between hemodynamic response variation and SCR variation. This work shows that early-emerging social reticence is associated with differences in emotion response convergence between hemodynamic and autonomic responses to extinguished threat cues. Children with high levels of social reticence exhibited strong SCR-brain coupling to both threat and safety cues, potentially reflecting momentary reinstatement of the context in which they initially experienced threatening stimuli.

## Interventions Based on Convergence Findings in Anxiety

The potential therapeutic application of SCR-brain convergence is highlighted by recent work on unconscious neural reinforcement approaches, like *Decoded Neurofeedback*, to treat anxiety. This approach aims to alter maladaptive autonomic patterns by reinforcing desirable brain activity, while bypassing the subjective unpleasantness in conscious exposure and attenuating attrition from treatment (Taschereau-Dumouchel et al., 2018; 2020). The emerging data demonstrate that autonomic fear responses (i.e., SCR) can be reduced by targeting specific neural representations associated with feared stimuli through closed-loop fMRI neural reinforcement. In one paradigm (Taschereau-Dumouchel et al., 2018), autonomic responses to specific, feared animals were reduced by pairing rewards with the unconscious activation of their neural representations. The participants, unaware of the specific animal being targeted, learned to regulate their brain activity, leading to reduced autonomic reactivity in response to these feared stimuli. This approach takes advantage of SCR-brain coupling by modulating autonomic responses through targeted, unconscious brain activity changes. Importantly, this procedure can be conducted without conscious exposure to the feared object, which can prevent the aversive distress typically associated with such exposure and affect drop-out rates. Of note, decoded neurofeedback does not directly counteract hyper-awareness but instead addresses the maladaptive neural representations of autonomic responses. Given the observed differences in convergence between SCR and brain activity in anxiety, such interventions may help recalibrate the relationship between autonomic responses and their subjective interpretation, potentially aiding in symptom reduction.

Ultimately, rather than examining overall emotion response convergence, studying the coupling between specific, neuroanatomically informed subsystems promises to inform etiological models of IP and accelerate innovations in intervention. This distinction aligns with the need to consider developmental trajectories and neural-autonomic convergence to better tailor treatments for pediatric psychopathology, particularly given the increasing burden of these mental health conditions in youth.

### Externalizing Problems

If IPs are rooted in anticipatory fear and worry, EPs are phenotypically expressed as anger, impulsivity, and inattention. Childhood EPs usually take the form of overt disruptive behaviors such as aggression, defiance, and hyperactivity. Children with EP show greater expression of negative affect, particularly anger (Campbell et al., 1986; Cole et al., 1994; Greenberg et al., 1991; Hastings et al., 2007; Raine et al., 1995), elevated emotional reactivity (Graziano & Garcia, 2016), and disrupted emotion regulation (Halligan et al., 2013; Marsh et al., 2008).

Anger, unlike fear, is typically associated with fight responses and a readiness to confront perceived threats. In typically developing children and adults, anger is often accompanied by increased autonomic activity, including elevated RSA and heart rate (Larsen et al., 2008; Stemmler, 2004). However, despite reporting high levels of anger (Wakschlag et al., 2007), many youth with EP exhibit reduced autonomic reactivity, particularly to aversive stimuli (Fairchild et al., 2008; Glenn et al., 2007; Kimonis et al., 2017; Michalska, Zeffiro et al., 2016). This pattern of autonomic hypo-arousal is a well-documented correlate of aggressive behavior (Raine, 2002), which may function not only to harm others (Parke & Slaby, 1983), but also to regulate under-arousal (Raine, 2002; Van Goozen et al., 2007). In these youth, low heart rate, SCRs, and SCLs often coexist with elevated negative affect (e.g., anger), distinguishing them from anxious youth, who exhibit hyper-awareness and heightened threat sensitivity.

Available research in a large sample of adolescents over-represented for EP used hierarchical linear modeling to examine emotion response convergence between heart rate changes and subjectively reported emotions in response to videos selected to elicit sadness, fear, anger, and happiness (Hastings et al., 2009). Results showed some emotion response convergence for sadness and fear: youth reporting more intense fear had elevated heart rate, whereas sadness intensity was inversely associated with heart rate. Of note, youth with EP displayed emotion response divergence—cardiac arousal was unrelated to their reported emotional intensity. This suggests that while they report feeling frightened by scary scenarios or saddened by others’ distress, they may lack the corresponding cardiac arousal that motivates adaptive responses, such as empathic concern. These findings align with other research linking EP to reduced convergence during sadness (Marsh et al., 2008) and to diminished empathic sadness (Blair, 1997; Michalska, Zeffiro et al., 2016). More broadly, a lack of alignment between subjective and autonomic responses to distressing stimuli may contribute to EP development. Increased SNS response signals a potential threat of harm to the self, thereby deterring aggression (Blair, 1995; Blair et al., 1997). Children who report anger or fear without corresponding autonomic activation (i.e., hypo-arousal) may miss bodily signals of threat and may be less likely to regulate their anger or aggressive impulses (Blair et al., 1997), helping explain the outbursts and risk-taking often seen in EP (Hastings et al., 2009).

Most research has focused on associations between individual autonomic measures and children's concurrent subjective emotional experiences. Findings show that autonomic indicators sometimes diverge from self-reported negative emotions in children with IP and EP. In IP—especially anxiety—children tend to self-report higher levels of distress than indicated by autonomic arousal (e.g., SCR), potentially reinforcing symptoms through heightening self-focus and overestimating threat. In EP, autonomic responses such as PNS-linked cardiac reactivity may be decoupled from self-reported anger or fear, potentially masking somatic threat cues. However, other recent findings suggest this pattern is not uniform: some high-anxious children imply increased emotion response convergence during middle childhood, with stronger alignment between subjective experience and autonomic arousal (Michalska et al., 2022). Rather than contradicting previous work, this suggests that emotion response convergence may be context-dependent—shaped by the type of anxiety, nature of the emotional stimulus, or developmental stage. These observations have implications for both theory and treatment. They urge us to refine our understanding of emotional processes and consider how treatments engage subjective vs autonomic components of emotion. Many current interventions target either autonomic reactivity or self-reported experience, but not both (LeDoux & Pine, 2016). Novel approaches—such as those aimed at implicit neural circuits (e.g., Taschereau-Dumouchel et al., 2017)—seek to bypass the discomfort of conscious exposure and may be especially promising for treatment-seeking anxious children. Build on these approaches, future efforts might intentionally target one emotional system while monitoring changes in the other, as in EFT or anxiolytic medication.

A different pattern from those described above for IP and EP is hypo-awareness, whereby typical or high levels of autonomic arousal are accompanied by lower self-reported emotions (e.g., Borelli et al., 2018). In contrast with hyper-awareness, hypo-awareness suggests suppression or diminished awareness of (mostly aversive) bodily states, which can lead to emotional avoidance, compromised memory function, and reduced social rapport (Borelli et al., 2018; Butler et al., 2003; Gross & Levenson, 1993, 1997; Richards & Gross, 2000). For example, youth suffering from post-traumatic stress disorder (PTSD) symptoms may attempt to numb or avoid engaging with negative sensations linked to traumatic events (e.g., Moore et al., 2008), thereby impeding recovery following trauma (Bonanno et al., 2004). As PTSD does not fit squarely within internalizing and externalizing dimensions, more work is needed to explore the role of emotion response divergence in developmental psychopathology more broadly.

## Summary of Emotion Response Convergence Across Development in Internalizing Problems and Externalizing Problems

An emergent empirical evidence base links individual differences in emotion response convergence to internalizing and externalizing symptoms across development. Some data suggest convergence strengthens with age, though this varies by context and system, with cardiac measures showing greater stability than electrodermal ones. Broadly, children with IP exhibit hyper-awareness, whereby their self-reported negative emotions, such as anticipatory fear or sadness, align with or even exceed their levels of autonomic arousal, potentially worsening symptoms. In contrast, children with EP exhibit hypo-arousal, with reduced autonomic responses but typical or elevated self-reports of anger. Overall, emotion response convergence may serve as a complex yet informative marker for developmental psychopathology.

## Challenges to Assumptions Underlying Emotion Response Convergence Research

Recent research has begun to examine how emotional subsystems relate to one another and how best to measure their associations, yet our understanding of emotion response convergence remains limited. In this final section, we argue that meaningful progress depends on addressing key assumptions about why different measures of emotional reactivity are expected to align. We highlight three such assumptions, discuss how addressing them can advance the field, and outline implications for both adaptive and maladaptive emotional development.

*Assumption #1. Emotional systems are symmetrically interrelated.* Given accumulating evidence for implicit/non-conscious emotions (Winkielman et al., 2005; Winkielman & Berridge, 2004), we generally assume that if an autonomic response can occur during an emotional episode in the absence of a subjective emotional experience (Murphy et al., 2018), such as elevated heart rate following an unfamiliar sound or subliminal facial expressions, then the inverse must also be true, i.e., that a subjective emotional experience can occur in the absence of an autonomic response. There is ample support for the former claim but far less for the latter (see section on EP). A case in point is the case of alexithymia, defined as difficulty identifying and describing one's own emotions (Nemiah, 1976). Individuals with alexithymia atypically represent affective interoceptive states: they are less accurate than typical individuals in perceiving their heart rate, sometimes not being aware of it at all (Shah et al., 2016) and exhibit atypical activation of the insula relative to those without alexithymia in response to visceral stimulation (Kano et al., 2007). Of note, such “dissociations” between autonomic and subjective emotional experience are frequently directional: alexithymics have autonomic responses without emotional awareness, but they do not report having feelings without concomitant autonomic reactions. Other than the scant examples in some youth with EP, we know of few other instances where this is the case unless someone is being deceptive. The reasons for this asymmetric “dissociation” (i.e., more documented autonomic responses to affective stimuli in the absence of a subjective emotional experience than the reverse) are not fully clear. However, it is likely easier to be unaware that one is having an emotional experience than it is to report an emotional experience in the absence of an autonomic reaction. Ultimately, it may be misleading to assume that the associations between implicit autonomic reactions and explicit subjective self-report must be symmetrical when instead, there may be constraints on the extent to which subjective emotional experiences can occur in the absence of autonomic or somatic responses. We are not arguing for an exclusively bottom-up model of emotion; brain regions involved in emotional responding are highly bidirectionally connected structures with continual access to information about the state of the body (Barrett & Bliss-Moreau, 2009). However, we contend that to characterize emotion response convergence more comprehensively, we need to test both directions of any hypothesized dissociations. As such, it would be informative if scholars collecting subjective self-report also collected concurrent autonomic data, when possible. Without access to the requisite equipment, researchers might consider including measures of interoceptive awareness or perceived autonomic activation (see e.g., Mauss et al., 2004).

*Assumption #2. Emotion response convergence during passive viewing is equivalent to emotion response convergence during active behavior.* This assumption might be flawed, given the functionalist view that emotions evolved to drive survival-relevant actions (Dickinson & Dearing, 2014; Frijda et al., 1989; Frijda & Parrott, 2011). If “feeling is for doing” (Nelissen & Zeelenberg, 2007), can we expect emotion response convergence in the absence of an action? Convergence across autonomic, cognitive, and behavioral systems likely exists to support coordinated action—not as an end in itself. Yet, most emotion research relies on passive paradigms, like threat conditioning tasks where participants simply view visual stimuli while autonomic, self-report, and hemodynamic measures are recorded. In real-world contexts, people might confront or actively avoid the impeding threat. Despite robust findings linking autonomic responses such as SCR to aversive states (Britton et al., 2013; Glenn et al., 2020; Michalska et al., 2017; Michalska, Shechner et al., 2016; Shechner et al., 2018), evidence for emotion response convergence across emotional systems in these passive settings is mixed (Michalska et al., 2019; Shechner et al., 2018). Critically, these paradigms require no coordinated action—successful conditioning occurs even with entirely implicit responses. But in real-life threat situations, behavioral responses are necessary and cannot be implicit—one cannot run away unconsciously. To evaluate whether emotion response convergence generalizes across passive and active tasks, we must test introducing active behavioral responses alters convergence patterns. Few studies have used autonomic measures, such as RSA and SCR to examine active behavior—a notable gap. Emerging work, however, suggests promise: FPS—an amplified startle reflex in the presence of threat cues—has been linked to avoidance decisions in fear extinction paradigms (van Meurs et al., 2014), showing that greater FPS predicts greater likelihood of shock avoidance. Moving forward, studies should directly compare emotion response convergence during passive tasks with those involving active behavioral engagement, such as decision-making games or virtual reality.

*Assumption #3. Self-report is an accurate readout of our internal state.* As the studies reviewed here show, emotion response convergence between autonomic and self-report measures of emotion is rare. One assumption underlying these observations is that what a person says is an accurate “readout” of their internal state. But this assumption, which equates the autonomic and behavioral with the mental, warrants further scrutiny. Autonomic, behavioral, and subjective systems may operate independently, and individuals often monitor and regulate their emotions, disrupting potential alignment (Dan-Glauser & Gross, 2013). Different regulation strategies can selectively affect physiology, subjective report, or behavior, influencing emotion response convergence (Butler et al., 2014; Dan-Glauser & Gross, 2013; Lanteigne et al., 2014; Mauss et al., 2011; Shiota & Levenson, 2012). Accordingly, emotion response convergence may vary depending on the strategy used.

Cultural and contextual factors also shape emotion expression. Display rules differ across relationships, situations, and cultures, impacting whether emotions are outwardly expressed (Cole et al., 2002; Cordaro et al., 2018; English et al., 2017; Matsumoto & Kupperbusch, 2001; Yeh et al., 2017). For instance, Americans tend to show what they feel, while Japanese individuals often suppress expression, weakening the link between felt and displayed emotion (Matsumoto et al., 1988). Similar patterns emerge in children: U.S. children appraise difficult interpersonal situations (e.g., falling and getting dirty in front of friends) with anger, while Nepalese children appraise them in terms of shame (Cole et al., 2002). These differences may reflect cultural norms learned early in development; by middle childhood, children already show awareness of display rules (Chung & Asher, 1996). However, socialization pressures vary across cultures, shaping emotional awareness and expression in nuanced ways across development. Future research should investigate how emotion response convergence interacts with sociocultural dynamics like emotion regulation values and interdependent self-construal (see Kraus & Kitayama, 2019). Since cultures differ in their endorsement of emotion suppression, exploring children's alignment with these norms could yield important insight into motivational and cultural influences on emotion response convergence.

In sum, reconsidering these three assumptions might have important theoretical and methodological consequences for the study of emotion response convergence.

## Conclusions and Future Challenges

Our goal in this review was to illustrate how convergence between sympathetic and parasympathetic activity and subjective self-report offers richer insight into developmental psychopathology than examining each system in isolation. Neuroimaging methods like fMRI complement autonomic measures by capturing real-time emotional experience and revealing dissociations between psychological processes often presumed to overlap. Nonetheless, key challenges remain. First, researchers must be deliberate in selecting autonomic measures, as emotional responses engage multiple, partly distinct systems linked to different neural substrates. Ignoring this complexity may obscure important individual differences in psychopathology risk. Second, advancing the field requires questioning assumptions about why and when emotional responses should converge: what functions does convergence serve and what behaviors do emotions drive? Third, we call for carefully designed, prospective longitudinal studies to characterize how emotion response convergence changes with development. Understanding when and how convergence occurs demands attention to variability across individuals, contexts, and time.

**Box 1.**
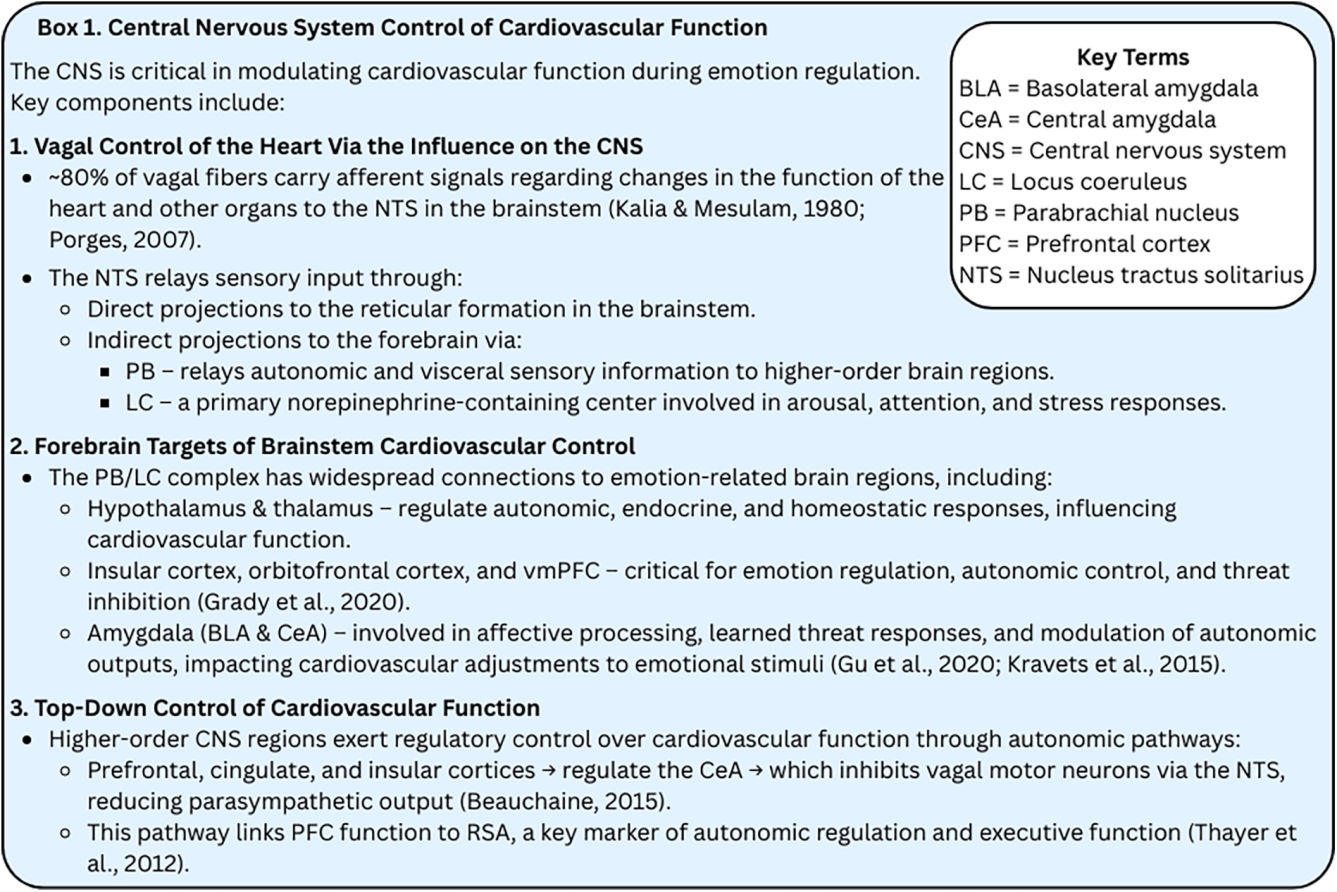
Central Nervous System Control of Cardiovascular Function.
